# Supplementary material for: The Childhood Cancer Diagnosis (CCD) Study: a UK observational study to describe referral pathways and quantify diagnostic intervals in children and young people with cancer
Source: BMJ Open. 2022 Feb 16;12(2):e058744. doi: 10.1136/bmjopen-2021-058744 (PMC8852751; doi:10.1136/bmjopen-2021-058744)
Supplement: Supplementary data [file bmjopen-2021-058744supp001.pdf]

Table S1. Eligible tumour types

Main Diagnostic Group	Subgroup
**I. Leukaemia**	(a) Lymphoid leukaemia<br>(b) Acute myeloid leukaemia<br>(c) Chronic myeloproliferative diseases<br>(d) Myelodysplastic syndrome or other myeloproliferative diseases<br>(e) Other
**II. Lymphoma & related**	(a) Hodgkin lymphoma<br>(b) Non-Hodgkin lymphoma except Burkitt lymphoma<br>(c) Burkitt lymphoma<br>(d) Lymphoreticular<br>(e) Other
**III. CNS tumour** [1]	(a) Ependymoma and choroid plexus tumour<br>(a.1) Ependymoma<br>(a.2) Choroid plexus tumours<br>• Papilloma<br>• Atypical papilloma<br>• Carcinoma<br>(b) Astrocytoma<br>• Pilocytic<br>• Subependymal giant cell<br>• Gliofibroma<br>• Protoplasmic<br>• Gemistocytic<br>• Fibrillary<br>• Pleomorphic xanthoastrocytoma<br>• Pilomyxoid<br>• Anaplastic<br>• Glioblastoma<br>• Unspecified (optic nerve)<br>• Unspecified (other sites)<br>(c) Intracranial and intraspinal embryonal tumour<br>(c.1) Medulloblastoma<br>• Desmoplastic/nodular/extensive nodularity<br>• Medullomyoblastoma<br>• Large cell/anaplastic<br>• Unspecified

[1] Stiller, C.A., Bayne, A.M., Chakrabarty, A. *et al.* Incidence of childhood CNS tumours in Britain and variation in rates by definition of malignant behaviour: population-based study. *BMC Cancer* **19,** 139 (2019). https://doi.org/10.1186/s12885-019-5344-7

Main Diagnostic Group	Subgroup
	(c.2) PNET<br>(c.3) Medulloepithelioma/neuroepithelioma<br>(c.4) Atypical teratoid/rhabdoid tumour<br><br>(d) Other glioma<br>(d.1) Oligodendroglioma<br>(d.2) Mixed and unspecified gliomas<br>• Mixed<br>• Angiocentric glioma<br>• Unspecified<br>(d.3) Other neuroepithelial tumours<br>• Gliomatosis cerebri<br>• Papillary tumour of the pineal region<br><br>(e) Other specified intracranial and intraspinal neoplasms<br>(e.1) Pituitary adenoma<br>(e.2) Craniopharyngioma<br>(e.3) Pineal parenchymal tumours<br>• Pineocytoma<br>• Pineoblastoma incl. PTID<br>(e.4) Mixed glial-neuronal tumours<br>• Desmoplastic infantile astrocytoma<br>• Dysembryoplastic neuroepithelial tumour<br>• Ganglioglioma<br>• Central neurocytoma<br>• Papillary glioneuronal tumour<br>• Gangliocytoma<br>(e.5) Meningioma<br><br>(f) Unspecified tumours<br><br>** Please note that germ cell tumours should be coded under Xa
**IV. Neuroblastoma**	(a) Neuroblastoma and ganglioneuroblastoma<br>(b) Other peripheral nervous cell tumour

Main Diagnostic Group	Subgroup
**V. Retinoblastoma**	--
**VI. Renal tumour**	(a) Nephroblastoma and other nonepithelial renal tumour<br>(a.1) Nephroblastoma (Wilms tumour)<br>(a.2) Rhabdoid renal tumour<br>(a.3) Kidney sarcoma<br>• CCS clear cell sarcoma (8964)<br>• Other<br>(a.4) pPNET of kidney<br>(b) Renal carcinoma<br>(c) Unspecified malignant renal tumour
**VII. Hepatic tumour**	(a) Hepatoblastoma<br>(b) Hepatic carcinoma<br>(c) Unspecified malignant hepatic tumour
**VIII. Bone tumour**	(a) Osteosarcomas<br>(b) Chondrosarcomas<br>(c) Ewing tumour and related sarcomas of bone<br>• Ewing tumour<br>• Askin tumour of bone<br>• pPNET of bone<br>(d) Other specified malignant bone tumour<br>(e) Unspecified malignant bone tumour
**IX. Soft tissue sarcoma**	(a) Rhabdomyosarcomas<br>(b) Fibrosarcomas, peripheral nerve sheath tumour, and other fibrous neoplasms (Non-rhabdomyosarcoma soft tissue sarcomas NRSTS)<br>(c) Kaposi sarcoma<br>(d) Other specified soft tissue sarcomas<br>(e) Unspecified soft tissue sarcomas
**X. Germ cell tumour**	(a) Intracranial and intraspinal germ cell tumours<br>(b) Malignant extracranial and extragonadal germ cell tumour

Main Diagnostic Group	Subgroup
	(c) Malignant gonadal germ cell tumour<br>(d) Gonadal carcinoma<br>(e) Other and unspecified malignant gonadal tumour
**XI. Carcinoma & melanoma**	(a) Adrenocortical carcinomas<br>(b) Thyroid carcinomas<br>(c) Nasopharyngeal carcinomas<br>(d) Malignant melanomas<br>(e) Skin carcinomas<br>(f) Other and unspecified carcinomas
**XII. Other & unspecified malignant**	(a) Other specified malignant tumour<br>(a.1) Gastrointestinal stromal tumour<br>(a.2) Pancreatoblastoma<br>(a.3) Pulmonary blastoma and pleuropulmonary blastoma<br>(a.4) Other complex mixed and stromal neoplasms<br>(a.5) Mesothelioma<br>(a.6) Other specified malignant tumour<br>(b) Other unspecified malignant tumour
