# Supplementary material for: The Childhood Cancer Diagnosis (CCD) Study: a UK observational study to describe referral pathways and quantify diagnostic intervals in children and young people with cancer
Source: BMJ Open. 2022 Feb 16;12(2):e058744. doi: 10.1136/bmjopen-2021-058744 (PMC8852751; doi:10.1136/bmjopen-2021-058744)
Supplement: Supplementary data [file bmjopen-2021-058744supp003.pdf]

Childhood Cancer Diagnosis Study Principal Investigator List

Aberdeen	Dr Hugh Bishop
Belfast	Dr Robert Johnston
Birmingham	Prof Bruce Morland
Bristol	Dr Rachel Dommett
Cambridge	Dr James Nicholson
Cardiff	Dr Melanie Adams
Edinburgh	Dr Mark Brougham
GOSH	Karen Howe
Glasgow	Dr Jairam Sastry
Leeds	Dr Christopher Lethaby
Leicester	Dr Emma Ross
Liverpool	Dr Katherine Cooper
Manchester	Dr Anthony Penn
Newcastle	Dr Tasnim Arif
Nottingham	Dr Madhumita Dandapani
Oxford	Dr Amy Mitchell
Royal Marsden	Dr Julia Chisholm
Sheffield	Dr Vicki Lee
Southampton	Jessica Bate
UCLH	Rachel Windsor
