# Supplementary material for: The Childhood Cancer Diagnosis (CCD) Study: a UK observational study to describe referral pathways and quantify diagnostic intervals in children and young people with cancer
Source: BMJ Open. 2022 Feb 16;12(2):e058744. doi: 10.1136/bmjopen-2021-058744 (PMC8852751; doi:10.1136/bmjopen-2021-058744)
Supplement: Supplementary data [file bmjopen-2021-058744supp002.pdf]

Participant study number: ____________________

THE CHILDHOOD CANCER DIAGNOSIS STUDY

Gender: ☐ Male ☐ Female **Ethnicity:** ______________________________

Age: ______years ______ months **Year of birth (YYYY):** ____________________

Diagnosis: ______________________

Tumour location: __________________ **Laterality (if applicable):** L / R / Midline/ Bilateral

Tumour stage: ____________________ **Clinical risk group (if applicable):** ____________

Key dates (DD/MM/YYYY)

- Date of symptom onset: ____________________________ ☐ Not known
- Date of first presentation to healthcare: _________________ ☐ Not known
- Date of clinical diagnosis: ____________________________ ☐ Not known
- Date of imaging: ___________________________________ ☐ Not known
- Date of biopsy: _____________________________ ☐ Not known

Route to diagnosis

- Who was the first healthcare professional (HCP) they saw about these symptoms:

  ☐ GP ☐ Paediatric emergency doctor ☐ Paediatrician ☐ Dentist ☐ Pharmacist

  ☐ Optometrist ☐Nurse practitioner ☐ Health visitor ☐ School nurse

  ☐ Other (please specify __________________________)

- How many HCP visits before diagnosis? _________ or ☐ 1-3 ☐ 4-6 ☐ 7-9 ☐10+

- Patient's place of care when the investigation that identified the tumour was requested:

  ☐ Primary care ☐ Outpatient ☐ Inpatient ☐ A&E ☐ Other __________________

- Was this an incidental finding?

  ☐ No ☐ Yes - asymptomatic ☐ Yes -with non-specific symptoms

- What was the source of referral leading to diagnosis?

Emergency presentation (A&E)	☐ Self-referral ☐ GP referral ☐ Optician referral ☐ Dentist referral ☐ MIU/Walk In Centre/NHS 111 ☐ Emergency transfer from another hospital ☐ Other HCP (please specify) ____________________________
GP referral	☐ Two week wait ☐ Routine referral ☐ Urgent referral to general paediatrician ☐ Other ______________________________________

Please keep this form in patient's medical records once completed

CRF Childhood cancer diagnosis

Participant study number: ____________________

Other	☐ Active surveillance (please specify _________________________________)<br>☐ Diagnosed by another specialty (e.g. ENT) ☐ Other _________________

Symptoms at diagnosis (Please tick all that apply)

Head, face, throat and neck

- ☐ Headache
- ☐ Vomiting
- ☐ Seizures
- ☐ Fits
- ☐ Visual abnormalities
- ☐ Papilloedema
- ☐ Leukocoria
- ☐ Abnormal eye movements
- ☐ Hearing loss
- ☐ Earache
- ☐ Torticollis/head tilt/stiff neck
- ☐ Sore throat/hoarse voice
- ☐ Difficulty swallowing
- ☐ Swollen glands
- ☐ Lump/swelling in face, jaw and skull
- ☐ Limited mouth opening
- ☐ Abnormal facial movements

Chest and Abdomen

- ☐ Shortness of breath
- ☐ Lump/swelling in chest wall or armpits
- ☐ Chest wall pain/axillary pain
- ☐ Abdominal pain/discomfort
- ☐ Abdominal distention/mass
- ☐ Haematuria
- ☐ Blood in stool
- ☐ Change in bowel habit
- ☐ Difficulty passing urine

Bones and Joints

- ☐ Bone/joint swelling
- ☐ Bone/joint pain
- ☐ Limp or leg weakness
- ☐ Slow in recovery after injury to bone/joint

Growth and Development

- ☐ Developmental delay
- ☐ Deterioration in balance/walking/speech
- ☐ Slow growth
- ☐ Weight loss
- ☐ Loss of appetite
- ☐ Early or late puberty
- ☐ Lump/swelling in pelvis, testicle or breast
- ☐ Unexplained bleeding after sex or between periods

Other symptoms

- ☐ Pallor
- ☐ Changes to moles
- ☐ Excessive bleeding/bruising/petechiae
- ☐ Persistent/recurrent unexplained screaming in young children
- ☐ Multiple infections
- ☐ Tiredness or fatigue
- ☐ Fever
- ☐ Night sweats

Any other symptom not listed above:

Please keep this form in patient's medical records once completed

CRF Childhood cancer diagnosis
