# Supplementary material for: The Childhood Cancer Diagnosis (CCD) Study: a UK observational study to describe referral pathways and quantify diagnostic intervals in children and young people with cancer
Source: BMJ Open. 2022 Feb 16;12(2):e058744. doi: 10.1136/bmjopen-2021-058744 (PMC8852751; doi:10.1136/bmjopen-2021-058744)
Supplement: Author's manuscript [file bmjopen-2021-058744.draft_revisions.pdf]

BMJ Open is committed to open peer review. As part of this commitment we make the peer review history of every article we publish publicly available.

When an article is published we post the peer reviewers' comments and the authors' responses online. We also post the versions of the paper that were used during peer review. These are the versions that the peer review comments apply to.

The versions of the paper that follow are the versions that were submitted during the peer review process. They are not the versions of record or the final published versions. They should not be cited or distributed as the published version of this manuscript.

BMJ Open is an open access journal and the full, final, typeset and author-corrected version of record of the manuscript is available on our site with no access controls, subscription charges or pay-per-view fees (http://bmjopen.bmj.com).

If you have any questions on BMJ Open's open peer review process please email info.bmjopen@bmj.com

BMJ Open

The Childhood Cancer Diagnosis (CCD) Study: Protocol of a UK-wide observational study

Journal:	*BMJ Open*
Manuscript ID	bmjopen-2021-058744
Article Type:	Protocol
Date Submitted by the Author:	26-Oct-2021
Complete List of Authors:	Shanmugavadivel, Dhurgshaarna; University of Nottingham Faculty of Medicine and Health Sciences, Academic Unit of Population and Lifespan Sciences<br>Liu, Jo-Fen; University of Nottingham Faculty of Medicine and Health Sciences, Academic Unit of Population and Lifespan Sciences<br>Ball-Gamble, Ashley; Children's Cancer and Leukaemia Group,<br>Polanco, Angela; Children's Cancer and Leukaemia Group<br>Vedhara, Kavita; University of Nottingham Faculty of Medicine and Health Sciences, Academic Unit of Population and Lifespan Sciences<br>Walker, David; University of Nottingham, Children's Brain Tumour Research Centre<br>Ojha, Shalini; University of Nottingham, Division of Graduate Entry Medicine; University of Nottingham, Academic Division of Child Health
Keywords:	Paediatric oncology < PAEDIATRICS, Epidemiology < TROPICAL MEDICINE, Public health < INFECTIOUS DISEASES

SCHOLARONE™
Manuscripts

Title: The Childhood Cancer Diagnosis (CCD) Study: Protocol of a UK-wide observational study

Corresponding authors:

Dr Dhurgshaarna Shanmugavadivel

Academic Unit of Population and Lifespan Sciences

School of Medicine

University of Nottingham

E/E2066 Queens Medical Centre

Derby Road, Nottingham

NG7 2UH

Tel: 011582 30630

Email: mszds@exmail.nottingham.ac.uk

Orchid ID: http://orcid.org/0000-0002-1912-4543

For peer review only

Co-authors:

1. Jo-Fen Liu, Academic Unit of Population and Lifespan Sciences, School of Medicine, University of Nottingham. Nottingham, UK. Email: jo-fen.liu@nottingham.ac.uk; Orchid ID: https://orcid.org/0000-0001-5796-7878
2. Ashley Gamble, Children's Cancer and Leukaemia Group. Leicester, UK. Email: Ashley.gamble@cclg.org.uk; https://orcid.org/0000-0002-0708-0918
3. Dr Angela Polanco, Children's Cancer and Leukaemia Group. Leicester, UK. Email apolancojohnson@hotmail.com; Orchid ID: 0000-0002-4619-0773
4. Professor Kavita Vedhara, Academic Unit of Population and Lifespan Sciences, School of Medicine, University of Nottingham. Nottingham, UK. Email: kavita.vedhara@nottingham.ac.uk ; Orchid ID: 0000-0002-9940-7534
5. Professor David Walker, Children's Brain Tumour Research Centre, School of Medicine, University of Nottingham. Nottingham, UK. Email: msadw12@exmail.nottingham.ac.uk
6. Dr Shalini Ojha, Academic Unit of Population and Lifespan Sciences, School of Health Sciences, University of Nottingham, UK and Children's Hospital, University Hospitals of Derby and Burton NHS Trust, Derby, UK. Email: shalini.ojha@nottingham.ac.uk; Orchid ID: https://orcid.org/0000-0001-5668-4227

Keywords:

Paediatric Oncology, Epidemiology, Public Health

For peer review only

Word Count: 2910 words

For peer review only

ABSTRACT

Introduction: Childhood cancer is diagnosed in 400,000 children and young people (CYP) aged 0-19 years worldwide annualy. In the UK, the individual risk of cancer from birth to age 25 years is 1 in 180. The overall five-year survival rate is 84%. Tumour diagnoses are at a later stage and mortality is higher when compared to those in other parts of Europe. Many CYPs experience delays to diagnosis which may contribute to poor outcomes. This study aims to understand the current pathway of childhood cancer referrals and diagnosis and quantify diagnostic intervals in the UK.

Methods and analysis: This is a prospective multi-centre observational study including all tertiary childhood cancer treatment centres in the UK. CYP (0-18 years) with a new diagnosis of cancer over the study period will be invited to participate. Data will be collected at initial diagnosis and 5 years after diagnosis. Data will include demographic details, clinical symptoms, tumour location, stage, and clinical risk group. In addition, key diagnostic dates and referral routes will be collected to calculate the diagnostic intervals. At five-years' follow-up, data will be collected on refractory disease, relapse and one and five-year survival.

Population characteristics will be presented with descriptive analyses with further analyses stratified by age, geographical region and cancer type. Associations between diagnostic delay and risk factors will be explored using logistic regression and estimate crude and adjusted odds ratios (ORs) with 95% confidence intervals (CIs) will be presented. A p value of <0.05 will be considered statistically significant.

For peer review only

Ethics: The study has favourable opinion from the York and Humber, Leeds West REC (19/YH/0416).

Dissemination: Results will be presented at academic conferences, published in peer-reviewed journals and disseminated through public messaging in collaboration with our charity partners and through a national awareness campaign (ChildCancerSmart).

Study registration: researchregistry.com (researchregistry5313).

For peer review only

ARTICLE SUMMARY

Strengths and Limitations

- This is the first nation-wide study to map childhood cancer diagnostic pathways.
- It includes the whole spectrum of cancers in children and young people.
- It will collect social, demographic and clinical data prospectively to reduce recall bias and explore associations of diagnostic intervals with these characteristics.
- This study will map the childhood cancer diagnostic pathway in the UK and provide the information needed to improve outcomes in children and young people with cancer.
- This is intended to model methods applicable to other national initiatives as part of the WHO Global Child Cancer initiative where levelling up outcomes is the primary aim.

For peer review only

INTRODUCTION

Childhood cancer is diagnosed in 400,000 children and young people (CYP) aged 0-19 years worldwide annualy.[1] Contrary to popular belief, childhood cancer is not rare.[2] In the UK, the individual risk of cancer from birth to age 25 years is 1 in 180 with 1645 new cases in 0-14 year olds and 2110 new cases in 15-24 year olds diagnosed each year.[3] Importantly, the incidence of childhood cancer has increased by 15% since the 1990s with a slightly higher incidence in boys than girls (in under 15s: boys, 1 in 420; girls, 1 in 490).[3] Whilst genetic predispositions are well documented, no modifiable or preventable risk factors have been identified.[4]

Childhood cancer is also the largest illness cause of death in CYP globally, and in the UK, responsible for over 1 in 5 deaths among 0-15 year olds.[3] As such, in 2018, the World Health Organisation (WHO) identified childhood cancer as a global disease burden and launched the Global Initiative for Childhood Cancer aiming to improve survival rates to 60% by 2030, saving over 1 million lives.[5]

The overall five-year survival rate in the UK is 84% across all childhood cancers, a statistically significant increase from 77% in 2001.[3] The improving cure rates over past decades have been achieved by the introduction of expertly delivered, complex therapies. Despite this, the UK performance for stage distribution at diagnosis for multiple tumours and outcomes compares unfavourably to those in leading European countries and survival rates are worse than in other countries e.g., Iceland has a 90.1% five-year survival rate.[6 7 8] A possible cause for the poorer outcomes is delay in diagnosis, the reasons for which may be multifactorial.

Symptoms in children are often non-specific, mimicking more common ailments. Furthermore, the perceived rarity of childhood cancer means it is often not considered

For peer review only

as a diagnosis until there are multiple symptoms by which time the disease is at a more advanced stage. Despite a systematic review confirming that CYP experience delays to diagnosis[9], there is a dearth of research exploring how and why such delays occur.

In the absence of recognised modifiable risk factors or feasible screening strategies the most effective approach to improving patient outcomes is early diagnosis that may enable prompt, effective treatment. Childhood cancer survivors are left with long-term effects, or late effects, caused by either the cancer itself or its treatment.[10] Late effects include problems with growth, organ function, fertility, cognition, and academic achievement.[11] It has been reported that two-thirds of childhood cancer survivors will develop at least one late-onset therapy-related complication.[12] Delays in diagnosis add further avoidable disabilities and increased risk of local tumours needing more extensive surgery for example amputation versus bone preserving surgery, partial nephrectomy versus total nephrectomy or liver resection versus liver transplant. Furthermore, advanced disease requires more extensive radiation fields with greater volumes of tissue irradiation with attendant risk for impaired tissue growth, focal brain/ endocrine tissue damage and enhanced second tumour risk. Early diagnosis can therefore reduce mortality and morbidity from the cancer itself and from the intensive burden of the curative treatment required to treat more advanced stage disease.

Whilst it is recognised globally that early diagnosis is crucial and that delays in diagnosis occur, we need to understand the current diagnostic pathway patterns and identify areas of potential improvement to enable improved care.

AIMS AND OBJECTIVES

For peer review only

The aim of this study is to understand the diagnostic intervals and referral pathways for CYP diagnosed with childhood cancer in the UK. The study objectives are: In CYPs with a new diagnosis of childhood cancers

a. To determine the diagnostic intervals
b. To determine the route of referral
c. To analyse the differences in diagnostic intervals and routes of referral between cancer types, age of presentation, and geographical region
d. To explore the associations between diagnostic intervals and patient and disease characteristics

METHODS AND ANALYSIS

Study design and setting

This is a prospective multi-centre observational study including all tertiary childhood cancer treatment centres i.e., Principal Treatment Centres (PTC) for Paediatric Oncology and Haematology in the UK (Figure 1).

Participant eligibility

All CYP aged 0-18 years with a new diagnosis of childhood cancer over the study period will be invited to participate (Table 1).

For peer review only

Table 1. Criteria for participant inclusion and exclusion

**Inclusion criteria**	Children and young people at age 0-18 years<br>**AND**<br>A new diagnosis of a childhood cancer<br>**WITH**<br>— the ability for their parent/guardian to give informed consent if age of the child is less than 16 years of age<br>— **Or** the ability for the young person to give informed consent if 16-18 years of age<br>— **Or** a consultee/legal representative is available to provide an opinion/consent if the young person is aged 16-18 and is deemed to lack capacity to consent for themselves.
**Exclusion criteria**	Age at diagnosis over 18 years of age<br>Patient diagnosed with cancer outside the UK

Study procedures

CYP will be recruited from all PTCs across the UK. Recruitment will be supported by the Children's Cancer and Leukaemia Group (CCLG).

Recruitment

Eligible participants will be recruited following a confirmed diagnosis of any cancer at a PTC. In the UK, once the CYP is referred to the PTC with a diagnosis of cancer, they have a consultation with their oncology care team. During this first consultation a full history of the events leading up to the diagnosis is recorded. A member of the clinical care team will identify eligible participants at this consultation. Recruitment will occur

For peer review only

via two possible methods (A or B) (Figure 2) to maximise participation, provide flexibility to recruiters and potential participants, and give CYP the optimal opportunity to participate.

Informed consent

All participants will provide written informed consent.

Method A: In person consent via paper forms

Participants who are inpatients on hospital wards will be given the study information and opportunity to discuss participation with a researcher. A researcher will seek written informed consent after at least 24 hours of the participant having received the study information.

Method B: Consent using online forms

During the initial consultation, a study flyer including the study title, a brief explanation of the study, and contact details of the study team will be offered to potential participants. The participants can access the study website, read the information, discuss with the research team if they wish, and give written informed consent via the study website (www.cclg.org.uk/CCDStudy).

For peer review only

Both pathways will be followed in keeping with the principles of Good Clinical Practice.[13] Participation will be entirely voluntary, and treatment and care will not be affected by the decision. It will also be explained the participant can withdraw at any time, but attempts will be made to avoid this.

For <16-year-old CYP, consent will be obtained from the parent/guardian. The capacity of such participants to consent for themselves and their wishes will be considered. Those between 16 and 18 years of age can provide consent if they are deemed, by the consenting researcher, to fully understand what is proposed and weigh this information in deciding. Involvement of the parents in decision-making will be encouraged unless the young person objects to this involvement. For those 16-18 years old who lack capacity to consent a consultee or legal representative will be consulted and asked to consent in keeping with the Mental Capacity Act (England and Wales); the Adults with Incapacity (Scotland) Act or the Mental Capacity Act (Northern Ireland) 2016).

Data collection

Data will be collected after recruitment from the first consultation at the PTC when the initial cancer diagnosis is made. Further follow-up data will be collected 5 years after the initial diagnosis. All PTCs that treat children and young people with cancer across the UK, through our collaboration with the Children's Cancer and Leukaemia

For peer review only

Group (CCLG), will participate. Data will be collected on standardised case report forms (CRFs) (Supplementary file 1).

Data will include demographic details and characteristics such as gender, age, ethnicity, and deprivation index[14] (calculated from home postcode). Clinical signs and symptoms at diagnosis, tumour location, tumour stage and clinical risk group (if applicable) will be collected. The International Classification of Childhood Cancer (ICCC-3)[1] will be used to code the diagnoses and the Toronto Paediatric Cancer Stage Guideline will be used to record tumour stage. These classification systems were chosen as they are internationally accepted and will therefore allow comparison with other studies.

Data for calculating diagnostic intervals

To calculate the diagnostic intervals, three key dates will be collected: date of symptom onset, date of first presentation to healthcare, and date of diagnosis (clinical, imaging, biopsy). The date of symptom onset will be determined by the participant or their parent/guardian and will be as reported. The date of first presentation to healthcare will be defined as the first presentation to any healthcare service with signs/symptoms attributable to the tumour as reported by the participant or their parent/guardian. The date of diagnosis is defined as the day when the cancer diagnosis was established, clinically, radiologically, or histologically as recorded in the participants' medical records at the PTC. It will include the dates of clinical diagnosis, imaging, biopsy,

For peer review only

histopathology report and/or multi-disciplinary team meeting where the diagnosis was established.

Where exact dates, such as participant reported dates, cannot be established approximates will be used. If the date is specified to the nearest week, it will be assumed to be the Monday at the start of the week. If specified to the nearest month, it will be recorded as the first day of the month and if specified to the nearest season, it will be recorded as the first day of April for “spring”, July for “summer” or “mid-year”, October for “fall” or “autumn”. In winter, attempt to determine whether the diagnosis was “late in the year” (use December with the applicable year) or “early in year” (use January with the respective year). Missing dates will be recorded as 01/01/1900.

Route to referral

To map out the route of referral, five key pieces of information will be collected: the first healthcare professional that the participant consulted about relevant symptom(s); the number of healthcare visits between onset of symptoms and diagnosis; the patient's place of care when the investigation that identified the tumour was requested; whether the diagnosis is an incidental finding; and the source of referral leading to diagnosis.

Planned 5 year follow up

For peer review only

For all participants, 5 years following the diagnosis, data will be collected including information about refractory disease, relapse and one year and five-year survival.

Sample size and justification

This is an observational study of all incident cases of childhood cancer over a two-year period. There are 1645 new diagnoses of cancer in the under 15 age group each year in the UK, and 2110 new diagnoses in the 15-25 age group each year in the UK.[3] As we are studying the 0-18 age group, we anticipate approximately 2000 new cases per year. Over two years, this would be 4000 new cases. Based on our previous experience, with a 70% recruitment rate, we expect around 2800 cases in the study period.

Statistical Analysis

This is an observational study and therefore there will be no hypothesis testing. Descriptive analysis will be used to characterise the study population. Data will be presented as mean and standard deviations (SD) or median and interquartile range (IQR) for continuous data and as counts and percentages for categorical data.

Three diagnostic intervals (Table 2) will be calculated and reported as median (IQR) as defined in the literature.[15]

For peer review only

Table 2: Definitions for diagnostic intervals[15]

Diagnostic interval	Definition
Total Diagnostic Interval (TDI)	time from symptom onset to the time diagnosis was established
Patient Interval (PI)	time from symptom onset to the time of first consultation with a healthcare professional
Diagnostic Interval (DI)	time from first consultation with a health care professional to the time diagnosis was established

Further data analysis stratified by age, geographical region and cancer type will be performed. Student t-test, Chi-squared or Kruskal-Wallis tests will be used for comparison between groups as appropriate. In the absence of a standard definition of diagnostic delay, median and the 75th percentile will be used as the cut-offs to define 'delay' and 'long delay', respectively. Logistic regression will be used to assess the associations between diagnostic delay and potential risk factors and estimate crude and adjusted odds ratios (ORs) with 95% confidence intervals (CIs). For each diagnostic interval, variables which are clinically or socially relevant or reach the significant level ($p<0.05$) at univariate analysis will be considered in multivariate analyses; effect modification will also be explored as appropriate. All statistical analyses will be conducted using statistical software Stata 16 SE (StataCorp. 2019. College Station, TX: StataCorp LLC) and/or R studio (R. RStudio, PBC, Boston, MA). A p value of <0.05 will be considered statistically significant in all analyses.

For peer review only

Patient and public involvement

The study was designed in collaboration with our charity partner, the Children's Cancer and Leukaemia Group (CCLG) and our parent advisor (AP) who played a key role in shaping the proposal. AP was involved throughout the study design process, including the consent process, and reviewing the patient information leaflet materials. In addition to this, members of the Paediatric Oncology Reference Team (PORT) who are an independent body of parents with experience of childhood cancer, also advised on the study protocol, and revised patient-facing documents. Members of PORT sit on the National Cancer Research Institute's Children's Cancer and Leukaemia Study group and regularly advise on research studies.

Ethical approvals

The study was given a favourable opinion by York and Humber, Leeds West REC (19/YH/0416) on 27/02/2020 and will be conducted in accordance with the ethical principles that have their origin in the Declaration of Helsinki, 1996; the principles of Good Clinical Practice and the UK Department of Health Policy Framework for Health and Social Care, 2017.[13]

For peer review only

Protocol registration

This study has been registered on researchregistry.com (researchregistry5313).

DISCUSSION

This is the first national observational study to measure diagnostic intervals and referral pathways for CYP. The data obtained will allow us to understand the current picture of childhood cancer diagnosis across the UK and identify factors associated with diagnostic delays. It will highlight areas with need for improvement where targeted public health intervention or larger policy changes could be implemented to enable earlier diagnosis. The WHO global effort to improve survival rates by 2030 has led to an urgency to understand the current picture and drive change to meet this ambitious but achievable target.

This national observational study follows from the success of the UK HeadSmart, early diagnosis of brain tumours campaign.[16] The HeadSmart public and professional awareness campaign was launched in 2011 in the UK, aiming to raise awareness of the signs and symptoms of brain tumours in children due to the long diagnostic intervals. The campaign has been associated with a reduction in the total diagnostic interval from 14.4 weeks in in 2006 to 6.5 weeks in 2015.[16 17]

This experience, where the impact of the public and professional awareness campaign (www.headsmart.org.uk) was shown to accelerate brain tumour diagnosis, justifies

For peer review only - http://bmjopen.bmj.com/site/about/guidelines.xhtml

this project. It will generate evidence to better understand the current pathway of childhood cancer referrals and diagnosis and quantify diagnostic intervals in the UK. The study will primarily inform UK practice but be used as a model worldwide, as part of WHO global challenge to level up outcomes for children with cancer.

Strengths

This study will recruit from all PTCs in the UK. This network of PTCs will allow maximal national coverage and give every CYP with a new diagnosis of cancer to participate. As childhood cancer is not treated by other services in the UK, this study will represent the whole UK population.

The collected data will allow analyses of diagnostic intervals and referral routes as well as their associations with social and clinical characteristics such as age, tumour type, geographical location, and tumour stage at presentation. Furthermore, the five-year follow-up will enable analyses of associations between diagnostic intervals and refractory disease, relapse and 1-and 5-year survival providing insight into whether delays in diagnosis affect survival.

Limitations

For peer review only

The diagnostic intervals will be collected from dates obtained from CYP and their families. There is a possibility of recall bias. We aim to minimise this by ensuring that the data are collected on the CYP's first presentation at the PTC where a through clinical history is recorded routinely. Adequate training will be provided to each site and all reporting clinicians will be given advice on how to record these dates as accurately as possible. All other data including data on outcome will be collected prospectively thus maximising accuracy.

For peer review only

Dissemination of results

This study is a collaboration between the University of Nottingham and the Children's cancer and Leukaemia Group (CCLG). The results will be disseminated to healthcare professionals through conference presentations and peer-reviewed journal publications. In addition, the results of the study will inform health policy makers in the UK to design and implement referral pathways that are improved and informed by this evidence. The data will also be disseminated through public messaging, raising awareness of the signs and symptoms of childhood cancer through a national awareness campaign called Child Cancer Smart.

Conclusion

This study is the first nation-wide study to explore the diagnostic pathway for cancer in CYP across the UK. The results will inform and influence practice in the UK and in

other countries where similar studies will allow the global community to work together to achieve the ambitions of the WHO Global Initiative for Childhood Cancer and improve CYP's lives.

For peer review only

AUTHORS CONTRIBUTIONS

DS, SO, JFL and DW conceived the study. DS, JFL, DW, AP, SO and KV were involved with study design. DS drafted the manuscript which was revised by SO and reviewed and edited by all authors.

DATA STATEMENT

Data will be published and available upon reasonable request.

FUNDING

This work is supported by the National Institute of Health Research (NIHR), through a Doctoral Research Fellowship for Dr Shanmugavadivel, grant number (DRF-2018-11-ST2-055). It is also supported by The Children's Cancer and Leukaemia Group.

ACKNOWLEDGEMENTS

We would like to acknowledge all site Principal Investigators at the PTCs (Supplementary file 2). We are particularly grateful as many sites were set up by research teams who continued to recruit and collect data in the midst of the COVID-19 pandemic.

For peer review only

We would also like to acknowledge all the children, young people and their families who have taken the time and consideration to participate in the study.

COMPETING INTERESTS STATEMENT

The authors have no competing interests to declare.

For peer review only

Figure 1: A map of all Principal Treatment Centres in the UK courtesy of Children's Cancer and Leukaemia Group (CCLG).

Figure 2: Recruitment methodology for the study

REFERENCES

1. Steliarova-Foucher E, Colombet M, Ries LAG, et al. International incidence of childhood cancer, 2001-10: a population-based registry study. *Lancet Oncol* 2017;18(6):719-31. doi: 10.1016/s1470-2045(17)30186-9 [published Online First: 2017/04/16]

2. Walker DA. Helping GPs to diagnose children's cancer. *British Journal of General Practice* 2021;71(705):151-52. doi: 10.3399/bjgp21X715241

3. England PH. UK CTYA Cancer Statistics report. 2021

4. Little J. Epidemiology of Childhood Cancer (International Agency for Research in Cancer (IARC) 1999

5. Organisation WH. WHO Global Initiative for Childhood Cancer: an overview, 2020.

6. Pritchard-Jones K. Childhood cancer in Britain: incidence, survival and mortality. *Br J Cancer* 2007;96(12):1927. doi: 10.038/sj.bjc.6603800. Epub 2007 Jun 12.

7. Pritchard-Jones K, Graf N, van Tinteren H, et al. Evidence for a delay in diagnosis of Wilms' tumour in the UK compared with Germany: implications for primary care for children. *Arch Dis Child* 2016;101(5):417-20. doi: 10.1136/archdischild-2015-309212 [published Online First: 2016/03/08]

8. Gatta G, Corazziari I, Magnani C, et al. Childhood cancer survival in Europe. *Ann Oncol* 2003;14 Suppl 5:v119-27. doi: 10.1093/annonc/mdg755 [published Online First: 2003/12/20]

9. Lethaby CD, Picton S, Kinsey SE, et al. A systematic review of time to diagnosis in children and young adults with cancer. *Arch Dis Child* 2013;98(5):349-55. doi: 10.1136/archdischild-2012-303034 [published Online First: 2013/03/12]

10. Armstrong GT, Kawashima T, Leisenring W, et al. Aging and risk of severe, disabling, life-threatening, and fatal events in the childhood cancer survivor

For peer review only

study. *J Clin Oncol* 2014;32(12):1218-27. doi: 10.1200/jco.2013.51.1055 [published Online First: 2014/03/19]

11. Pediatrics AAo. Long-term follow-up care for pediatric cancer survivors. *Pediatrics* 2009;123(3):906-15. doi: 10.1542/peds.2008-3688 [published Online First: 2009/03/04]

12. Oeffinger KC, Mertens AC, Sklar CA, et al. Chronic health conditions in adult survivors of childhood cancer. *N Engl J Med* 2006;355(15):1572-82. doi: 10.1056/NEJMsa060185 [published Online First: 2006/10/13]

13. Authority HR. UK policy framework for health and social care research. 2017

14. Ministry of Housing CaLG. The English Indices of Deprivation. 2019

15. Weller D, Vedsted P, Rubin G, et al. The Aarhus statement: improving design and reporting of studies on early cancer diagnosis. *Br J Cancer* 2012;106(7):1262-7. doi: 10.1038/bjc.2012.68 [published Online First: 2012/03/15]

16. Aware HBBT, Aware HBBT, Walker D, et al. A new clinical guideline from the Royal College of Paediatrics and Child Health with a national awareness campaign accelerates brain tumor diagnosis in UK children—“HeadSmart: Be Brain Tumour Aware”. *Neuro-Oncology* 2015;18(3):445-54. doi: 10.1093/neuonc/nov187

17. Shanmugavadivel D, Liu JF, Murphy L, et al. Accelerating diagnosis for childhood brain tumours: an analysis of the HeadSmart UK population data. *Arch Dis Child* 2020;105(4):355-62. doi: 10.1136/archdischild-2018-315962 [published Online First: 2019/10/28]

For peer review only

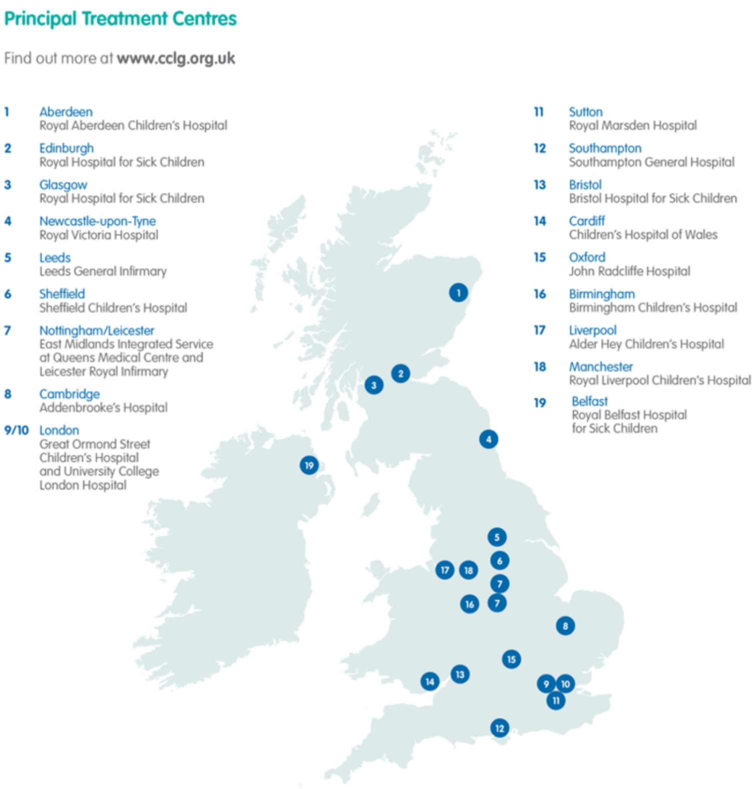

Figure 1: A map of all Principal Treatment Centres in the UK courtesy of Children's Cancer and Leukaemia Group (CCLG).

274x396mm (82 x 82 DPI)

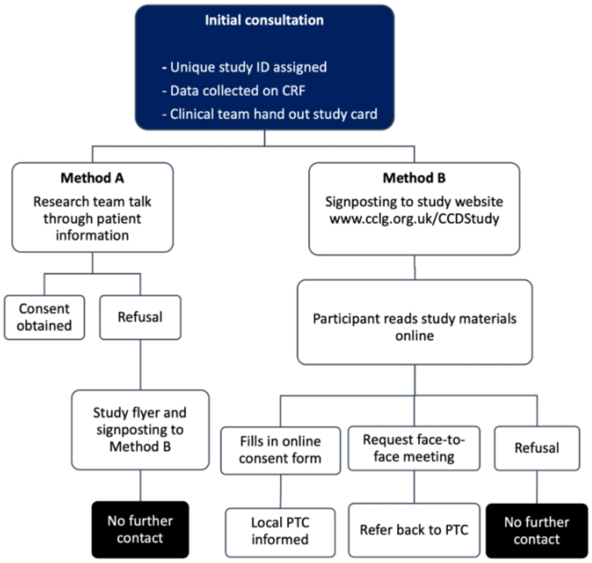

Figure 2: Recruitment methodology for the study

298x206mm (109 x 109 DPI)

Participant study number: ____________________

THE CHILDHOOD CANCER DIAGNOSIS STUDY

Gender: ☐ Male ☐ Female **Ethnicity:** ______________________________

Age: ______years ______ months **Year of birth (YYYY):** ______________________

Diagnosis: ______________________

Tumour location: ____________________ **Laterality (if applicable):** L / R / Midline/ Bilateral

Tumour stage: ______________________ **Clinical risk group (if applicable):** _____________

Key dates (DD/MM/YYYY)

- Date of symptom onset: ______________________________ ☐ Not known
- Date of first presentation to healthcare: __________________ ☐ Not known
- Date of clinical diagnosis: ______________________________ ☐ Not known
- Date of imaging: _____________________________________ ☐ Not known
- Date of biopsy/surgery: _________________________________ ☐ Not known

Route to diagnosis

- Who was the first healthcare professional (HCP) they saw about these symptoms:

  ☐ GP ☐ Paediatric emergency doctor ☐ Paediatrician ☐ Dentist ☐ Pharmacist

  ☐ Optometrist ☐Nurse practitioner ☐ Health visitor ☐ School nurse

  ☐ Other (please specify ___________________________)

- How many HCP visits before diagnosis? __________ or ☐ 1-3 ☐ 4-6 ☐ 7-9 ☐10+

- Patient's place of care when the investigation that identified the tumour was requested:

  ☐ Primary care ☐ Outpatient ☐ Inpatient ☐ A&E ☐ Other ___________________

- Was this an incidental finding?

  ☐ No ☐ Yes - asymptomatic ☐ Yes -with non-specific symptoms

- What was the source of referral leading to diagnosis?

Emergency presentation (A&E)	☐ Self-referral ☐ GP referral ☐ Optician referral ☐ Dentist referral ☐ MIU/Walk In Centre/NHS 111 ☐ Emergency transfer from another hospital ☐ Other HCP (please specify) ______________________________
GP referral	☐ Two week wait ☐ Routine referral ☐ Urgent referral to general paediatrician ☐ Other ____

Please keep this form in patient's medical records once completed

CRF Childhood cancer diagnosis v1.1 27.02.2020

For peer review only - http://bmjopen.bmj.com/site/about/guidelines.xhtml

Participant study number: ____________________

Other	☐ Active surveillance (please specify ________________________________)<br>☐ Diagnosed by another specialty (e.g. ENT) ☐ Other ________________

Symptoms at diagnosis (Please tick all that apply)

Head, face, throat and neck

- ☐ Headache
- ☐ Vomiting
- ☐ Seizures
- ☐ Fits
- ☐ Visual abnormalities
- ☐ Papilloedema
- ☐ Leukocoria
- ☐ Abnormal eye movements
- ☐ Hearing loss
- ☐ Earache
- ☐ Torticollis/head tilt/stiff neck
- ☐ Sore throat/hoarse voice
- ☐ Difficulty swallowing
- ☐ Swollen glands
- ☐ Lump/swelling in face, jaw and skull
- ☐ Limited mouth opening
- ☐ Abnormal facial movements

Chest and Abdomen

- ☐ Shortness of breath
- ☐ Lump/swelling in chest wall or armpits
- ☐ Chest wall pain/axillary pain
- ☐ Abdominal pain/discomfort
- ☐ Abdominal distention/mass
- ☐ Haematuria
- ☐ Blood in stool
- ☐ Change in bowel habit
- ☐ Difficulty passing urine

Bones and Joints

- ☐ Bone/joint swelling
- ☐ Bone/joint pain
- ☐ Limp or leg weakness
- ☐ Slow in recovery after injury to bone/joint

Growth and Development

- ☐ Developmental delay
- ☐ Deterioration in balance/walking/speech
- ☐ Slow growth
- ☐ Weight loss
- ☐ Loss of appetite
- ☐ Early or late puberty
- ☐ Lump/swelling in pelvis, testicle or breast
- ☐ Unexplained bleeding after sex or between periods

Other symptoms

- ☐ Pallor
- ☐ Changes to moles
- ☐ Excessive bleeding/bruising/petechiae
- ☐ Persistent/recurrent unexplained screaming in young children
- ☐ Multiple infections
- ☐ Tiredness or fatigue
- ☐ Fever
- ☐ Night sweats

Any other symptom not listed above:

Please keep this form in patient's medical records once completed

For peer review only - http://bmjopen.bmj.com/site/about/guidelines.xhtml

Childhood Cancer Diagnosis Study Principal Investigator List

Aberdeen	Dr Hugh Bishop
Belfast	Dr Robert Johnston
Birmingham	Prof Bruce Morland
Bristol	Dr Rachel Dommett
Cambridge	Dr James Nicholson
Cardiff	Dr Melanie Adams
Edinburgh	Dr Mark Brougham
GOSH	Karen Howe
Glasgow	Dr Jairam Sastry
Leeds	Dr Christopher Lethaby
Leicester	Dr Emma Ross
Liverpool	Dr Katherine Cooper
Manchester	Dr Anthony Penn
Newcastle	Dr Tasnim Arif
Nottingham	Dr Madhumita Dandapani
Oxford	Dr Amy Mitchell
Royal Marsden	Dr Julia Chisholm
Sheffield	Dr Vicki Lee
Southampton	Jessica Bate
UCLH	Rachel Windsor

For peer review only

The Childhood Cancer Diagnosis (CCD) Study: a UK observational study to describe referral pathways and quantify diagnostic intervals in children and young people with cancer.

Journal:	*BMJ Open*
Manuscript ID	bmjopen-2021-058744.R1
Article Type:	Protocol
Date Submitted by the Author:	14-Jan-2022

Complete List of Authors:	Shanmugavadivel, Dhurgshaarna; University of Nottingham Faculty of Medicine and Health Sciences, Academic Unit of Population and Lifespan Sciences<br>Liu, Jo-Fen; University of Nottingham Faculty of Medicine and Health Sciences, Academic Unit of Population and Lifespan Sciences<br>Ball-Gamble, Ashley; Children’s Cancer and Leukaemia Group,<br>Polanco, Angela; Children’s Cancer and Leukaemia Group<br>Vedhara, Kavita; University of Nottingham Faculty of Medicine and Health Sciences, Academic Unit of Population and Lifespan Sciences<br>Walker, David; University of Nottingham, Children's Brain Tumour Research Centre<br>Ojha, Shalini; University of Nottingham, Division of Graduate Entry Medicine; University of Nottingham, Academic Division of Child Health

**Primary Subject Heading**:	Paediatrics
Secondary Subject Heading:	Oncology, Public health, Epidemiology, Health policy
Keywords:	Paediatric oncology < PAEDIATRICS, Epidemiology < TROPICAL MEDICINE, Public health < INFECTIOUS DISEASES

Title: The Childhood Cancer Diagnosis (CCD) Study: a UK observational study to describe referral pathways and quantify diagnostic intervals in children and young people with cancer.

Corresponding authors:

Dr Dhurgshaarna Shanmugavadivel

Academic Unit of Population and Lifespan Sciences

School of Medicine

University of Nottingham

E/E2066 Queens Medical Centre

Derby Road, Nottingham

NG7 2UH

Tel: 011582 30630

Email: shaarnashan@doctors.org.uk

Orchid ID: http://orcid.org/0000-0002-1912-4543

For peer review only

Co-authors:

1. Jo-Fen Liu, Academic Unit of Population and Lifespan Sciences, School of Medicine, University of Nottingham. Nottingham, UK. Email: jo-fen.liu@nottingham.ac.uk; Orchid ID: https://orcid.org/0000-0001-5796-7878
2. Ashley Gamble, Children's Cancer and Leukaemia Group. Leicester, UK. Email: Ashley.gamble@cclg.org.uk; https://orcid.org/0000-0002-0708-0918
3. Dr Angela Polanco, Children's Cancer and Leukaemia Group. Leicester, UK. Email apolancojohnson@hotmail.com; Orchid ID: 0000-0002-4619-0773
4. Professor Kavita Vedhara, Centre for Academic Primary Care, Academic Unit of Population and Lifespan Sciences, School of Medicine, University of Nottingham. Nottingham, UK. Email: kavita.vedhara@nottingham.ac.uk ; Orchid ID: 0000-0002-9940-7534
5. Professor David Walker, Children's Brain Tumour Research Centre, School of Medicine, University of Nottingham. Nottingham, UK. Email: msadw12@exmail.nottingham.ac.uk
6. Dr Shalini Ojha, Academic Unit of Population and Lifespan Sciences, School of Health Sciences, University of Nottingham, UK and Children's Hospital, University Hospitals of Derby and Burton NHS Trust, Derby, UK. Email: shalini.ojha@nottingham.ac.uk; Orchid ID: https://orcid.org/0000-0001-5668-4227

Keywords:

Paediatric Oncology, Epidemiology, Public Health

Word Count: 3082 words

For peer review only

ABSTRACT

Introduction: Childhood cancer is diagnosed in 400,000 children and young people (CYP) aged 0-19 years worldwide annually. In the UK, a child's cumulative cancer risk increases from 1 in 4690 from birth to aged 1, to 1 in 470 by age 15. Once diagnosed, access to treatments offer survival to adulthood for over 80%. Tumour diagnoses are at a later stage and mortality is higher when compared to those in other parts of Europe. This means higher risk, more intensive therapies for a cure. Some CYPs are known to experience delays to diagnosis which may further contribute to poor outcomes. This study aims to understand the current pathway of childhood cancer referrals and diagnosis and quantify diagnostic intervals in the UK.

Methods and analysis: This is a prospective multi-centre observational study including all tertiary childhood cancer treatment centres in the UK. CYP (0-18 years) with a new diagnosis of cancer over the study period will be invited to participate. Data will be collected at initial diagnosis and 5 years after diagnosis. Data will include demographic details, clinical symptoms, tumour location, stage, and clinical risk group. In addition, key diagnostic dates and referral routes will be collected to calculate the diagnostic intervals. At five-years' follow-up, data will be collected on refractory disease, relapse and one and five-year survival.

Population characteristics will be presented with descriptive analyses with further analyses stratified by age, geographical region and cancer type. Associations

For peer review only

between diagnostic intervals/delay and risk factors will be explored using multiple regression and logistic regression.

Ethics: The study has favourable opinion from the York and Humber, Leeds West REC (19/YH/0416).

Dissemination: Results will be presented at academic conferences, published in peer-reviewed journals and disseminated through public messaging in collaboration with our charity partners through a national awareness campaign (ChildCancerSmart).

Study registration: researchregistry.com (researchregistry5313).

For peer review only

ARTICLE SUMMARY

Strengths and Limitations

- The first nation-wide study using prospective point of care data to map childhood cancer diagnostic pathways with measurements of diagnostic intervals.
- It includes the whole spectrum of cancers in children and young people aged 0-18.
- It will collect social, demographic and clinical data prospectively to reduce recall bias and explore associations of diagnostic intervals with these characteristics.
- Diagnostic interval will be calculated from the point of symptom onset. However, these data will necessarily be retrospective and may, therefore, be affected by recall bias.

For peer review only

INTRODUCTION

Childhood cancer is diagnosed in 400,000 children and young people (CYP) 0-19 years worldwide annually.[1] Contrary to popular belief, childhood cancer is not rare.[2] In the UK, the individual risk of cancer from birth to age 15 years is 1 in 470[2] with 1645 new cases in 0-14 year olds and 2110 new cases in 15-24 year olds diagnosed each year.[3] Importantly, the incidence of childhood cancer has increased by 15% since the 1990s with a slightly higher incidence in boys than girls (in under 15s: boys, 1 in 420; girls, 1 in 490).[3] Whilst genetic predispositions are well documented, no modifiable or preventable risk factors have been identified.[4]

Childhood cancer is also the largest illness cause of death in CYP globally, and in the UK, responsible for over 1 in 5 deaths among 0-15 year olds.[3] As such, in 2018, the World Health Organisation (WHO) identified childhood cancer as a global disease burden and launched the Global Initiative for Childhood Cancer aiming to improve survival rates to 60% by 2030, saving over 1 million lives.[5]

The overall five-year survival estimate in the UK is 84% across all childhood cancers, a statistically significant increase from 77% in 2001.[3] The improving cure rates over past decades have been achieved by the introduction of expertly delivered, complex therapies. Despite this, the UK performance for stage distribution at diagnosis for multiple tumours and outcomes compares unfavourably to those in leading European countries and survival rates are worse than in other countries e.g., Iceland has a 90.1% five-year survival rate.[6 7 8] A possible cause for the poorer outcomes is delay in diagnosis, the reasons for which may be multifactorial. Previous studies have reported diagnostic pathways for CYP with cancer in England using data reported to central registries.[9] Children (0-14) were found to present more commonly via an emergency

For peer review only

presentation than those aged 15-25 or 26-44, however this did not seem to cause a significant disadvantage in survival outcome.[9]

Symptoms in children are often non-specific, mimicking more common ailments. Furthermore, the perceived rarity of childhood cancer means it is often not considered as a diagnosis until there are multiple symptoms by which time the disease is at a more advanced stage. Despite a systematic review confirming that CYP experience delays to diagnosis[10], there is a dearth of research exploring how and why such delays occur.

In the absence of recognised modifiable risk factors or feasible screening strategies the most effective approach to improving patient outcomes is early diagnosis that may enable prompt, effective treatment. Childhood cancer survivors are left with long-term effects, or late effects, caused by either the cancer itself or its treatment.[11] Late effects include problems with growth, organ function, fertility, cognition, and academic achievement.[12] It has been reported that two-thirds of childhood cancer survivors will develop at least one late-onset therapy-related complication.[13] Delays in diagnosis add further avoidable disabilities and increased risk of local tumours needing more extensive surgery for example amputation versus bone preserving surgery, partial nephrectomy versus total nephrectomy or liver resection versus liver transplant. Furthermore, advanced disease requires more extensive radiation fields with greater volumes of tissue irradiation with attendant risk for impaired tissue growth, focal brain/ endocrine tissue damage and enhanced second tumour risk. Early diagnosis can therefore reduce mortality and morbidity from the cancer itself and from the intensive burden of the curative treatment required to treat more advanced stage disease.

For peer review only

Whilst it is recognised globally that early diagnosis is crucial and that delays in diagnosis occur, we need to understand the current diagnostic pathway patterns and identify areas of potential improvement to enable improved care.

AIMS AND OBJECTIVES

For peer review only

The aim of this study is to understand the diagnostic intervals and referral pathways for CYP diagnosed with childhood cancer in the UK. The study objectives are: In CYPs with a new diagnosis of childhood cancers

a. To determine the diagnostic intervals
b. To determine the route of referral
c. To analyse the differences in diagnostic intervals and routes of referral between cancer types, age of presentation, and geographical region
d. To explore the associations between diagnostic intervals and patient and disease characteristics

METHODS AND ANALYSIS

Study design and setting

This is a prospective multi-centre observational study including all tertiary childhood cancer treatment centres i.e., Principal Treatment Centres (PTC) for Paediatric Oncology and Haematology in the UK (Figure 1).

Participant eligibility

All CYP aged 0-18 years with a new diagnosis of childhood cancer over the study period will be invited to participate (Table 1). This age group was chosen to correlate with the CYP cared for by paediatric clinical services within the UK.

Table 1. Criteria for participant inclusion and exclusion

**Inclusion criteria**	Children and young people at age 0-18 years<br>**AND**<br>A new diagnosis of a childhood cancer (see Table S1 for complete list)<br>**WITH**<br>— the ability for their parent/guardian to give informed consent if age of the child is less than 16 years of age<br>— **Or** the ability for the young person to give informed consent if 16-18 years of age<br>— **Or** a consultee/legal representative is available to provide an opinion/consent if the young person is aged 16-18 and is deemed to lack capacity to consent for themselves.
**Exclusion criteria**	Age at diagnosis over 18 years of age<br>Patient diagnosed with cancer outside the UK

For peer review only

Study procedures

CYP will be recruited from all PTCs across the UK. Recruitment will be supported by the Children's Cancer and Leukaemia Group (CCLG) who have an established research network. The study opened to recruitment on 30th September 2020 and is on the National Institute of Health Research (NIHR) Portfolio.

Recruitment

Eligible participants will be recruited following a confirmed diagnosis of any cancer at a PTC. In the UK, once the CYP is referred to the PTC with a diagnosis of cancer, they have a consultation with their oncology care team. During this first consultation a full history of the events leading up to the diagnosis is recorded. A member of the clinical care team will identify eligible participants at this consultation. Recruitment will occur via two possible methods (A or B) (Figure 2) to maximise participation, provide flexibility to recruiters and potential participants, and give CYP the optimal opportunity to participate.

For peer review only

Informed consent

All participants will provide written informed consent.

Method A: In person consent via paper forms

Participants who are inpatients on hospital wards will be given the study information and opportunity to discuss participation with a researcher. A researcher will seek written informed consent after at least 24 hours of the participant having received the study information.

Method B: Consent using online forms

During the initial consultation, a study flyer including the study title, a brief explanation of the study, and contact details of the study team will be offered to potential participants. The participants can access the study website, read the information, discuss with the research team if they wish, and give written informed consent via the study website (www.cclg.org.uk/CCDStudy).

Both pathways will be followed in keeping with the principles of Good Clinical Practice.[14] Participation will be entirely voluntary, and treatment and care will not be affected by the decision. It will also be explained the participant can withdraw at any time, but attempts will be made to avoid this.

For <16-year-old CYP, consent will be obtained from the parent/guardian. Those between 16 and 18 years of age can provide consent. Involvement of the parents in decision-making will be encouraged unless the young person objects to this involvement. For those 16-18 years old who lack capacity to consent a consultee or

For peer review only

legal representative will be consulted and asked to consent in keeping with the Mental Capacity Act (England and Wales); the Adults with Incapacity (Scotland) Act or the Mental Capacity Act (Northern Ireland) 2016).

Data collection

Data will be collected by the clinical care team after recruitment from the first consultation at the PTC when the initial cancer diagnosis is made. Further follow-up data will be collected 5 years after the initial diagnosis. All PTCs that treat children and young people with cancer across the UK, through our collaboration with the Children's Cancer and Leukaemia Group (CCLG), will participate. Data will be collected on standardised case report forms (CRFs) (Supplementary file 1).

Data will include demographic details and characteristics such as sex, age, ethnicity, and Index of Multiple Deprivation (IMD)[15] (calculated from home postcode without any health domain component). Clinical signs and symptoms at diagnosis, tumour location, tumour stage and clinical risk group (if applicable) will be collected. The International Classification of Childhood Cancer (ICCC-3)[1] will be used to code the diagnoses and the Toronto Paediatric Cancer Stage Guideline will be used to record tumour stage. These classification systems were chosen as they are internationally accepted and will therefore allow comparison with other studies.

Primary outcome measure

For peer review only

The primary outcome measure is the Total Diagnostic Interval (TDI), as defined in the literature (Table 2).

Secondary outcome measures

The secondary outcome measures are the Patient Interval (PI) and the Diagnostic Interval (DI) (Table 2).

Table 2: Definitions for diagnostic intervals[16]

Diagnostic interval	Definition
Total Diagnostic Interval (TDI)	time from symptom onset to the time diagnosis was established (sum of PI and DI)
Patient Interval (PI)	time from symptom onset to the time of first consultation with a healthcare professional
Diagnostic Interval (DI)	time from first consultation with a health care professional to the time diagnosis was established

Data for calculating diagnostic intervals

To calculate the diagnostic intervals, three key dates will be collected: date of symptom onset, date of first presentation to healthcare, and date of diagnosis (clinical, imaging, biopsy). The date of symptom onset will be determined by the participant or their parent/guardian. Thus it will necessarily be retrospective and self-reported. The date

For peer review only

of first presentation to healthcare will be defined as the first presentation to any healthcare service with signs/symptoms attributable to the tumour as reported by the participant or their parent/guardian. The date of diagnosis is defined as the day when the cancer diagnosis was established, clinically, radiologically, or histologically as recorded in the participants' medical records at the PTC. It will include the dates of clinical diagnosis, imaging, biopsy, histopathology report and/or multi-disciplinary team meeting where the diagnosis was established.

Where exact dates, such as participant reported dates, cannot be established approximates will be used. If the date is specified to the nearest week, it will be assumed to be the Monday at the start of the week. If specified to the nearest month, it will be recorded as the first day of the month and if specified to the nearest season, it will be recorded as the first day of April for "spring", July for "summer" or "mid-year", October for "fall" or "autumn". In winter, attempt to determine whether the diagnosis was "late in the year" (use December with the applicable year) or "early in year" (use January with the respective year). Missing dates will be recorded as 01/01/1900.

Route to referral

To map out the route of referral, five key pieces of information will be collected: the first healthcare professional that the participant consulted about relevant symptom(s); the number of healthcare visits between onset of symptoms and diagnosis; the patient's place of care when the investigation that identified the tumour was requested;

For peer review only

whether the diagnosis is an incidental finding; and the source of referral leading to diagnosis.

Planned 5 year follow up

Centres will be approached at 5 years after the close to recruitment and asked to submit dates of first relapse, refractory illness (by date of pathology or imaging) and/or death to calculate 1- and 5-year survival. A separate follow up protocol will be written in due course.

Sample size and justification

This is an observational study of all incident cases of childhood cancer over a two-year period. There are 1645 new diagnoses of cancer in the under 15 age group each year in the UK, and 2110 new diagnoses in the 15-25 age group each year in the UK.[3] As we are studying the 0-18 age group, we anticipate approximately 2000 new cases per year. Over two years, this would be 4000 new cases. Based on our previous experience, with a 70% recruitment rate, we expect around 2800 cases in the study period.

Statistical Analysis

For peer review only

Descriptive analysis will be used to characterise the study population. Data will be presented as mean and standard deviations (SD) or median and interquartile range (IQR) for continuous data and as counts and percentages for categorical data.

Diagnostic intervals

The three diagnostic intervals (TDI, PI and DI) will be calculated and reported as median (IQR) as defined in the literature.[16]

Sub-group analyses stratified by age, sex, geographical region, socio-economic status and cancer type will be performed. Student t-test, Chi-squared or Kruskal-Wallis tests will be used for comparison between groups as appropriate.

Clinical factors of interest are tumour type, tumour location and presentation symptom. Outcome variable of interest is diagnostic intervals (as contentious) and diagnostic delay (diagnostics intervals categorised using percentile cut points). Multiple regression and multivariate logistic regression will be used to estimate adjusted regression coefficients and adjusted odds ratios for each clinical factor, respectively. Univariate and full clinical model will be fitted, and relationships of all variables including will also be assessed in order to select the variables to be included in the final parsimonious adjusted model. Effect modification with socio-demographic factors (age, sex, geographical regions, socio-economic status as represented by IMD calculated by resident postcode as a categorical variable) will be explored as appropriate.

All statistical analyses will be conducted using statistical software Stata 16 SE (StataCorp. 2019. College Station, TX: StataCorp LLC) and/or R studio (R. RStudio,

For peer review only

PBC, Boston, MA). A p value of <0.05 will be considered statistically significant in all analyses.

Missing data

Where two or more key dates are missing despite these measures, the participant will not be included in the diagnostic interval analysis. The number of such participants will be reported in the study flowchart and summary statistics comparing participants who had missing data with those whose full data-set were available will also be reported. We will not be doing any multiple imputation. For each analysis, numbers missing will be reported.

Patient and public involvement

The study was designed in collaboration with our charity partner, the Children's Cancer and Leukaemia Group (CCLG) and our parent advisor (AP) who played a key role in shaping the proposal. AP was involved throughout the study design process, including the consent process, and reviewing the patient information leaflet materials. In addition to this, members of the Paediatric Oncology Reference Team (PORT) who are an independent body of parents with experience of childhood cancer, also advised on the study protocol, and revised patient-facing documents. Members of PORT sit on the National Cancer Research Institute's Children's Cancer and Leukaemia Study group and regularly advise on research studies.

For peer review only

Ethical approvals

The study was given a favourable opinion by York and Humber, Leeds West REC (19/YH/0416) on 27/02/2020 and will be conducted in accordance with the ethical principles that have their origin in the Declaration of Helsinki, 1996; the principles of Good Clinical Practice and the UK Department of Health Policy Framework for Health and Social Care, 2017.[14]

Protocol registration

This study has been registered on researchregistry.com (researchregistry5313).

DISCUSSION

This is the first national observational study to measure diagnostic intervals and referral pathways for CYP. The data obtained will allow us to understand the current picture of childhood cancer diagnosis across the UK and identify factors associated with diagnostic delays. It will highlight areas with need for improvement where targeted public health interventions or larger policy changes could be implemented to enable earlier diagnosis. The WHO global effort to improve survival rates by 2030 has led to

For peer review only

an urgency to understand the current picture and drive change to meet this ambitious but achievable target.

This national observational study follows from the success of the UK HeadSmart, early diagnosis of brain tumours campaign.[17] The HeadSmart public and professional awareness campaign was launched in 2011 in the UK, aiming to raise awareness of the signs and symptoms of brain tumours in children due to the long diagnostic intervals. The campaign has been associated with a reduction in the total diagnostic interval from 14.4 weeks in in 2006 to 6.5 weeks in 2015.[17 18]

This experience, where the impact of the public and professional awareness campaign (www.headsmart.org.uk) was shown to accelerate brain tumour diagnosis, justifies this project. It will generate evidence to better understand the current pathway of childhood cancer referrals and diagnosis and quantify diagnostic intervals in the UK. The study will primarily inform UK practice but be used as a model worldwide, as part of WHO global challenge to level up outcomes for children with cancer.

Strengths

This study will recruit from all PTCs in the UK. This network of PTCs will allow maximal national coverage and give every CYP with a new diagnosis of cancer to participate. As childhood cancer is not treated by other services in the UK, this study will represent the whole UK population.

For peer review only

The collected data will allow analyses of diagnostic intervals and referral routes as well as their associations with social and clinical characteristics such as age, tumour type, geographical location, and tumour stage at presentation. Furthermore, the five-year follow-up will enable analyses of associations between diagnostic intervals and refractory disease, relapse and 1-and 5-year survival providing insight into whether delays in diagnosis affect survival.

For peer review only

Limitations

The diagnostic intervals will be collected from dates obtained from CYP and their families. There is a possibility of recall bias. We aim to minimise this by ensuring that the data are collected on the CYP's first presentation at the PTC where a thorough clinical history is recorded routinely. Adequate training will be provided to each site and all reporting clinicians will be given advice on how to record these dates as accurately as possible. All other data including data on outcome will be collected prospectively thus maximising accuracy.

Dissemination of results

This study is a collaboration between the University of Nottingham and the Children's cancer and Leukaemia Group (CCLG). The results will be disseminated to healthcare professionals through conference presentations and peer-reviewed journal publications. In addition, the results of the study will inform health policy makers in the

UK to design and implement referral pathways that are improved and informed by this evidence. The data will also be disseminated through public messaging, raising awareness of the signs and symptoms of childhood cancer through a national awareness campaign called Child Cancer Smart.

For peer review only

AUTHORS CONTRIBUTIONS

DS, SO, JFL and DW conceived the study. DS, JFL, DW, AP, SO and KV were involved with study design. DS drafted the manuscript which was revised by SO and reviewed and edited by all authors.

DATA STATEMENT

Data will be published and available upon reasonable request.

FUNDING

This work is supported by the National Institute of Health Research (NIHR), through a Doctoral Research Fellowship for Dr Shanmugavadivel, grant number (DRF-2018-11-ST2-055). It is also supported by The Children’s Cancer and Leukaemia Group, award number (CCLG CCS 001/ CCLG CCS 002).

ACKNOWLEDGEMENTS

We would like to acknowledge all site Principal Investigators at the PTCs (Supplementary file 2). We are particularly grateful as many sites were set up by

For peer review only

research teams who continued to recruit and collect data in the midst of the COVID-19 pandemic.

We would also like to acknowledge all the children, young people and their families who have taken the time and consideration to participate in the study.

COMPETING INTERESTS STATEMENT

The authors have no competing interests to declare.

Figure 1: A map of all Principal Treatment Centres in the UK courtesy of Children's Cancer and Leukaemia Group (CCLG).

Figure 2: Recruitment methodology for the study

REFERENCES

For peer review only

1. Steliarova-Foucher E, Colombet M, Ries LAG, et al. International incidence of childhood cancer, 2001-10: a population-based registry study. *Lancet Oncol* 2017;18(6):719-31. doi: 10.1016/s1470-2045(17)30186-9 [published Online First: 2017/04/16]

2. Walker DA. Helping GPs to diagnose children's cancer. *British Journal of General Practice* 2021;71(705):151-52. doi: 10.3399/bjgp21X715241

3. England PH. UK CTYA Cancer Statistics report. 2021

4. Little J. Epidemiology of Childhood Cancer (International Agency for Research in Cancer (IARC) 1999

5. Organisation WH. WHO Global Initiative for Childhood Cancer: an overview, 2020.

6. Pritchard-Jones K. Childhood cancer in Britain: incidence, survival and mortality. *Br J Cancer* 2007;96(12):1927. doi: 10.038/sj.bjc.6603800. Epub 2007 Jun 12.

7. Pritchard-Jones K, Graf N, van Tinteren H, et al. Evidence for a delay in diagnosis of Wilms' tumour in the UK compared with Germany: implications for primary care for children. *Arch Dis Child* 2016;101(5):417-20. doi: 10.1136/archdischild-2015-309212 [published Online First: 2016/03/08]

8. Gatta G, Corazziari I, Magnani C, et al. Childhood cancer survival in Europe. *Ann Oncol* 2003;14 Suppl 5:v119-27. doi: 10.1093/annonc/mdg755 [published Online First: 2003/12/20]

9. L. Irvine CS, R. Dommet, G. Lyratzopoulos, R. Feltbower, A. Herbert, S. Sandhu, S. Winters, S. Wilne, L. Elliss-Brookes, M. McCabe. FP076 SIOP19-0842 Diagnostic Pathways for Children Diagnosed with Cancer in England.

For peer review only

Pediatric Blood & Cancer 2019;66(S4):e27989. doi: https://doi.org/10.1002/pbc.27989

10. Lethaby CD, Picton S, Kinsey SE, et al. A systematic review of time to diagnosis in children and young adults with cancer. *Arch Dis Child* 2013;98(5):349-55. doi: 10.1136/archdischild-2012-303034 [published Online First: 2013/03/12]
11. Armstrong GT, Kawashima T, Leisenring W, et al. Aging and risk of severe, disabling, life-threatening, and fatal events in the childhood cancer survivor study. *J Clin Oncol* 2014;32(12):1218-27. doi: 10.1200/jco.2013.51.1055 [published Online First: 2014/03/19]
12. Pediatrics AAo. Long-term follow-up care for pediatric cancer survivors. *Pediatrics* 2009;123(3):906-15. doi: 10.1542/peds.2008-3688 [published Online First: 2009/03/04]
13. Oeffinger KC, Mertens AC, Sklar CA, et al. Chronic health conditions in adult survivors of childhood cancer. *N Engl J Med* 2006;355(15):1572-82. doi: 10.1056/NEJMsa060185 [published Online First: 2006/10/13]
14. Authority HR. UK policy framework for health and social care research. 2017
15. Ministry of Housing CaLG. The English Indices of Deprivation. 2019
16. Weller D, Vedsted P, Rubin G, et al. The Aarhus statement: improving design and reporting of studies on early cancer diagnosis. *Br J Cancer* 2012;106(7):1262-7. doi: 10.1038/bjc.2012.68 [published Online First: 2012/03/15]
17. Aware HBBT, Aware HBBT, Walker D, et al. A new clinical guideline from the Royal College of Paediatrics and Child Health with a national awareness campaign accelerates brain tumor diagnosis in UK children—"HeadSmart: Be Brain Tumour Aware". *Neuro-Oncology* 2015;18(3):445-54. doi: 10.1093/neuonc/nov187
18. Shanmugavadivel D, Liu JF, Murphy L, et al. Accelerating diagnosis for childhood brain tumours: an analysis of the HeadSmart UK population data. *Arch Dis Child* 2020;105(4):355-62. doi: 10.1136/archdischild-2018-315962 [published Online First: 2019/10/28]

For peer review only

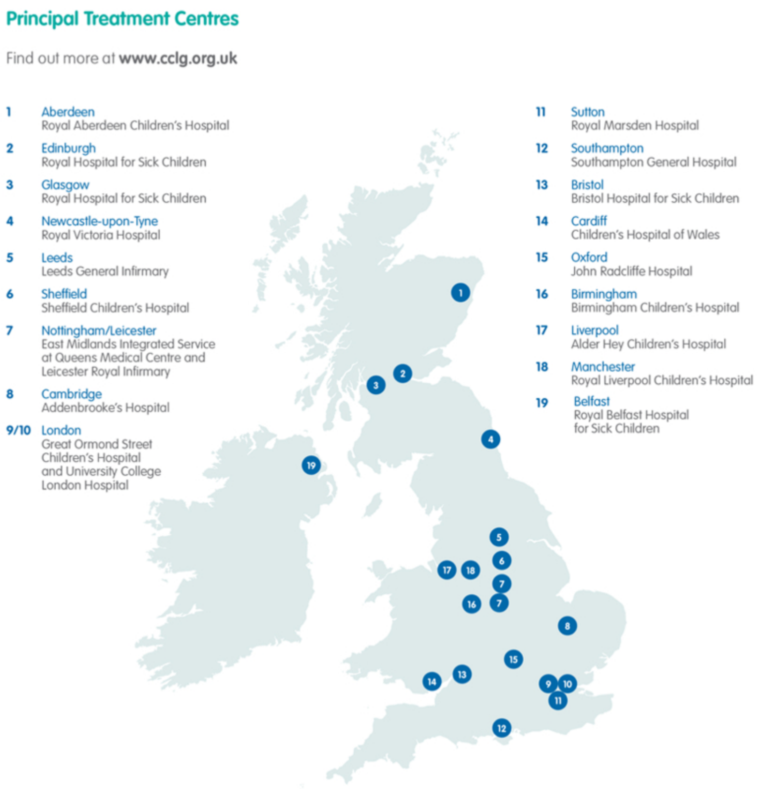

Figure 1: A map of all Principal Treatment Centres in the UK courtesy of Children's Cancer and Leukaemia Group (CCLG).

274x396mm (82 x 82 DPI)

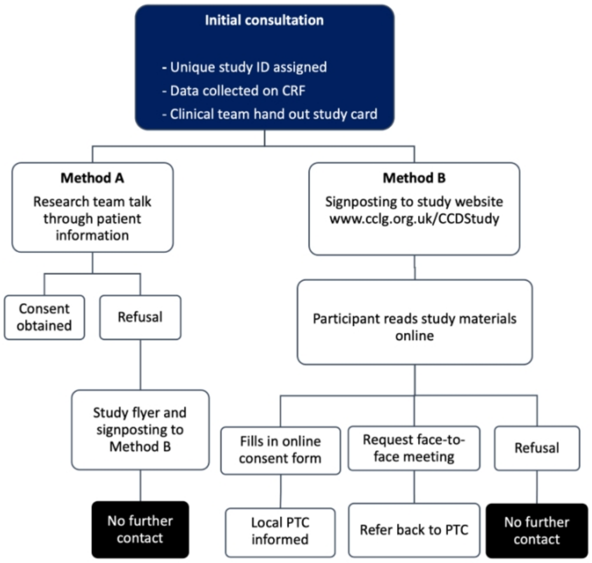

Figure 2: Recruitment methodology for the study

298x206mm (109 x 109 DPI)

Table S1. Eligible tumour types

Main Diagnostic Group	Subgroup
**I. Leukaemia**	(a) Lymphoid leukaemia<br>(b) Acute myeloid leukaemia<br>(c) Chronic myeloproliferative diseases<br>(d) Myelodysplastic syndrome or other myeloproliferative diseases<br>(e) Other
**II. Lymphoma & related**	(a) Hodgkin lymphoma<br>(b) Non-Hodgkin lymphoma except Burkitt lymphoma<br>(c) Burkitt lymphoma<br>(d) Lymphoreticular<br>(e) Other
**III. CNS tumour [1]**	(a) Ependymoma and choroid plexus tumour<br>(a.1) Ependymoma<br>(a.2) Choroid plexus tumours<br>• Papilloma<br>• Atypical papilloma<br>• Carcinoma<br>(b) Astrocytoma<br>• Pilocytic<br>• Subependymal giant cell<br>• Gliofibroma<br>• Protoplasmic<br>• Gemistocytic<br>• Fibrillary<br>• Pleomorphic xanthoastrocytoma<br>• Pilomyxoid<br>• Anaplastic<br>• Glioblastoma<br>• Unspecified (optic nerve)<br>• Unspecified (other sites)<br>(c) Intracranial and intraspinal embryonal tumour<br>(c.1) Medulloblastoma<br>• Desmoplastic/nodular/extensive nodularity<br>• Medullomyoblastoma<br>• Large cell/anaplastic<br>• Unspecified

[1] Stiller, C.A., Bayne, A.M., Chakrabarty, A. *et al.* Incidence of childhood CNS tumours in Britain and variation in rates by definition of malignant behaviour: population-based study. *BMC Cancer* **19,** 139 (2019). https://doi.org/10.1186/s12885-019-5344-7

For peer review only

Main Diagnostic Group	Subgroup
	(c.2) PNET<br>(c.3) Medulloepithelioma/neuroepithelioma<br>(c.4) Atypical teratoid/rhabdoid tumour<br><br>(d) Other glioma<br>(d.1) Oligodendroglioma<br>(d.2) Mixed and unspecified gliomas<br>• Mixed<br>• Angiocentric glioma<br>• Unspecified<br>(d.3) Other neuroepithelial tumours<br>• Gliomatosis cerebri<br>• Papillary tumour of the pineal region<br><br>(e) Other specified intracranial and intraspinal neoplasms<br>(e.1) Pituitary adenoma<br>(e.2) Craniopharyngioma<br>(e.3) Pineal parenchymal tumours<br>• Pineocytoma<br>• Pineoblastoma incl. PTID<br>(e.4) Mixed glial-neuronal tumours<br>• Desmoplastic infantile astrocytoma<br>• Dysembryoplastic neuroepithelial tumour<br>• Ganglioglioma<br>• Central neurocytoma<br>• Papillary glioneuronal tumour<br>• Gangliocytoma<br>(e.5) Meningioma<br><br>(f) Unspecified tumours<br><br>** Please note that germ cell tumours should be coded under Xa
**IV. Neuroblastoma**	(a) Neuroblastoma and ganglioneuroblastoma<br>(b) Other peripheral nervous cell tumour

For peer review only

Main Diagnostic Group	Subgroup
**V. Retinoblastoma**	--
**VI. Renal tumour**	(a) Nephroblastoma and other nonepithelial renal tumour<br>(a.1) Nephroblastoma (Wilms tumour)<br>(a.2) Rhabdoid renal tumour<br>(a.3) Kidney sarcoma<br>• CCS clear cell sarcoma (8964)<br>• Other<br>(a.4) pPNET of kidney<br>(b) Renal carcinoma<br>(c) Unspecified malignant renal tumour
**VII. Hepatic tumour**	(a) Hepatoblastoma<br>(b) Hepatic carcinoma<br>(c) Unspecified malignant hepatic tumour
**VIII. Bone tumour**	(a) Osteosarcomas<br>(b) Chondrosarcomas<br>(c) Ewing tumour and related sarcomas of bone<br>• Ewing tumour<br>• Askin tumour of bone<br>• pPNET of bone<br>(d) Other specified malignant bone tumour<br>(e) Unspecified malignant bone tumour
**IX. Soft tissue sarcoma**	(a) Rhabdomyosarcomas<br>(b) Fibrosarcomas, peripheral nerve sheath tumour, and other fibrous neoplasms (Non-rhabdomyosarcoma soft tissue sarcomas NRSTS)<br>(c) Kaposi sarcoma<br>(d) Other specified soft tissue sarcomas<br>(e) Unspecified soft tissue sarcomas
**X. Germ cell tumour**	(a) Intracranial and intraspinal germ cell tumours<br>(b) Malignant extracranial and extragonadal germ cell tumour

For peer review only

Main Diagnostic Group	Subgroup
	(c) Malignant gonadal germ cell tumour<br>(d) Gonadal carcinoma<br>(e) Other and unspecified malignant gonadal tumour
**XI. Carcinoma & melanoma**	(a) Adrenocortical carcinomas<br>(b) Thyroid carcinomas<br>(c) Nasopharyngeal carcinomas<br>(d) Malignant melanomas<br>(e) Skin carcinomas<br>(f) Other and unspecified carcinomas
**XII. Other & unspecified malignant**	(a) Other specified malignant tumour<br>(a.1) Gastrointestinal stromal tumour<br>(a.2) Pancreatoblastoma<br>(a.3) Pulmonary blastoma and pleuropulmonary blastoma<br>(a.4) Other complex mixed and stromal neoplasms<br>(a.5) Mesothelioma<br>(a.6) Other specified malignant tumour<br>(b) Other unspecified malignant tumour

For peer review only

Participant study number: ____________________

THE CHILDHOOD CANCER DIAGNOSIS STUDY

Gender: ☐ Male ☐ Female **Ethnicity:** ______________________________

Age: ______years ______ months **Year of birth (YYYY):** ______________________

Diagnosis: ______________________

Tumour location: __________________ **Laterality (if applicable):** L / R / Midline/ Bilateral

Tumour stage: ____________________ **Clinical risk group (if applicable):** ____________

Key dates (DD/MM/YYYY)

- Date of symptom onset: ___________________________ ☐ Not known
- Date of first presentation to healthcare: ________________ ☐ Not known
- Date of clinical diagnosis: ___________________________ ☐ Not known
- Date of imaging: __________________________________ ☐ Not known
- Date of biopsy: _____________________________ ☐ Not known

Route to diagnosis

- Who was the first healthcare professional (HCP) they saw about these symptoms:

  ☐ GP ☐ Paediatric emergency doctor ☐ Paediatrician ☐ Dentist ☐ Pharmacist

  ☐ Optometrist ☐Nurse practitioner ☐ Health visitor ☐ School nurse

  ☐ Other (please specify _________________________)

- How many HCP visits before diagnosis? _________ or ☐ 1-3 ☐ 4-6 ☐ 7-9 ☐10+

- Patient's place of care when the investigation that identified the tumour was requested:

  ☐ Primary care ☐ Outpatient ☐ Inpatient ☐ A&E ☐ Other _________________

- Was this an incidental finding?

  ☐ No ☐ Yes - asymptomatic ☐ Yes -with non-specific symptoms

- What was the source of referral leading to diagnosis?

Emergency presentation (A&E)	☐ Self-referral ☐ GP referral ☐ Optician referral ☐ Dentist referral ☐ MIU/Walk In Centre/NHS 111 ☐ Emergency transfer from another hospital ☐ Other HCP (please specify) ___________________________
GP referral	☐ Two week wait ☐ Routine referral ☐ Urgent referral to general paediatrician ☐ Other ____________________________________

Please keep this form in patient's medical records once completed

CRF Childhood cancer diagnosis

For peer review only - http://bmjopen.bmj.com/site/about/guidelines.xhtml

For peer review only

Participant study number: ____________________

Other	☐ Active surveillance (please specify ________________________________)<br>☐ Diagnosed by another specialty (e.g. ENT) ☐ Other _________________

Symptoms at diagnosis (Please tick all that apply)

Head, face, throat and neck

☐ Headache
☐ Vomiting
☐ Seizures
☐ Fits
☐ Visual abnormalities
☐ Papilloedema
☐ Leukocoria
☐ Abnormal eye movements
☐ Hearing loss
☐ Earache
☐ Torticollis/head tilt/stiff neck
☐ Sore throat/hoarse voice
☐ Difficulty swallowing
☐ Swollen glands
☐ Lump/swelling in face, jaw and skull
☐ Limited mouth opening
☐ Abnormal facial movements

Chest and Abdomen

☐ Shortness of breath
☐ Lump/swelling in chest wall or armpits
☐ Chest wall pain/axillary pain
☐ Abdominal pain/discomfort
☐ Abdominal distention/mass
☐ Haematuria
☐ Blood in stool
☐ Change in bowel habit
☐ Difficulty passing urine

Bones and Joints

☐ Bone/joint swelling
☐ Bone/joint pain
☐ Limp or leg weakness
☐ Slow in recovery after injury to bone/joint

Growth and Development

☐ Developmental delay
☐ Deterioration in balance/walking/speech
☐ Slow growth
☐ Weight loss
☐ Loss of appetite
☐ Early or late puberty
☐ Lump/swelling in pelvis, testicle or breast
☐ Unexplained bleeding after sex or between periods

Other symptoms

☐ Pallor
☐ Changes to moles
☐ Excessive bleeding/bruising/petechiae
☐ Persistent/recurrent unexplained screaming in young children
☐ Multiple infections
☐ Tiredness or fatigue
☐ Fever
☐ Night sweats

Any other symptom not listed above:

Please keep this form in patient's medical records once completed

For peer review only - http://bmjopen.bmj.com/site/about/guidelines.xhtml
For peer review only

Childhood Cancer Diagnosis Study Principal Investigator List

Aberdeen	Dr Hugh Bishop
Belfast	Dr Robert Johnston
Birmingham	Prof Bruce Morland
Bristol	Dr Rachel Dommett
Cambridge	Dr James Nicholson
Cardiff	Dr Melanie Adams
Edinburgh	Dr Mark Brougham
GOSH	Karen Howe
Glasgow	Dr Jairam Sastry
Leeds	Dr Christopher Lethaby
Leicester	Dr Emma Ross
Liverpool	Dr Katherine Cooper
Manchester	Dr Anthony Penn
Newcastle	Dr Tasnim Arif
Nottingham	Dr Madhumita Dandapani
Oxford	Dr Amy Mitchell
Royal Marsden	Dr Julia Chisholm
Sheffield	Dr Vicki Lee
Southampton	Jessica Bate
UCLH	Rachel Windsor

For peer review only
